# QM/MM Studies of Contemporary and Novel Membrane Raft Fluorescent Probes

**DOI:** 10.3390/molecules190710230

**Published:** 2014-07-15

**Authors:** Hannah L. Blake, David Robinson

**Affiliations:** School of Chemistry, University of Nottingham, University Park, Nottingham NG7 2RD, UK; E-Mail: david.robinson@nottingham.ac.uk

**Keywords:** QM/MM, fluorescence, membrane probes, TDDFT, lipid raft

## Abstract

We have studied a number of contemporary and novel membrane probes, selected for their structural similarity to membrane raft components, in order to properly anchor themselves within a sphingolipid/cholesterol rich region. A QM/MM approach was adopted in order to understand the structural and electrostatic influences of fluorescence emission shifts of the probes in different lipid and solvation environments. The proposed modifications to the membrane probes have shown encouraging data relating not only to emission shifts within the membrane, but also their ability to anchor within a membrane raft domain and the stability to internalization within a membrane system.

## 1. Introduction

Hybrid quantum mechanics/molecular mechanics (QM/MM) has revolutionized the study of small molecules in solvated and complex biological environments [1]. Among the varied applications of QM/MM, the prediction of electronic spectra is a useful approach. Within this approach, one usually chooses the chromophore (or fluorophore) of interest to be described with a QM method (e.g., DFT), while describing the surrounding environment with the less computationally demanding molecular mechanics. In particular, for absorption spectra, we usually optimize the ground-state geometry of the molecule and find the vertical transition energy. The adiabatic excitation energy (often referred to as the 0-0 transition) can be found by also optimizing the geometry at the excited state of interest. Once we have this geometry, a vertical excitation energy calculation gives us the energy gap that is equivalent to the energy of fluorescence.

Fluorescent molecules are widely used as probes of membrane function by the membrane biophysics community [2,3,4,5,6,7,8,9,10,11,12,13,14]. Most natural biomolecules found within membranes possess few or no optical moieties which can easily be exploited and so the introduction of an external fluorescently labelled molecule is necessary for imaging *in vitro*. There are many different probes, of which two are widely used. The first type, the aminonaphthylethenylpyridinium (ANEP) dyes, are fast potentiometric membrane probes that can yield fluorescence shifts in response to changes in the membrane dipole potential, Ψ_d_, in the sub-millisecond regime [11,15,16,17,18,19,20,21,22,23]. One particular ANEP dye, di-8-ANEPPS, has been shown both experimentally and theoretically to be sensitive only to Ψ_d_ and not to explicit molecular interactions [11,23]. Di-8-ANEPPS is also being exploited for non-linear optical imaging, potentially offering more information on the membrane environment being reported on [24]. The second type of probe are the BODIPY probes [25,26,27], which have found use in a wide range of applications, including as the lasing medium in a dye laser [28,29,30], biological imaging [31,32,33], organic light-emitting diodes [34], energy transfer cassettes [35], potential photosensitizers in photodynamic therapy [36] and dye-sensitized solar cells [37]. BODIPY dyes have emission shifts that are sensitive to solvent polarisability or solvent polarity [38,39,40,41], and that they are substantially less sensitive to hydrogen-bonding [41]. Our previous study of the core BODIPY molecule demonstrated a slight deviation from planarity of the geometry in the emitting state, using both CASPT2 and DFT [14]. Subsequent studies have found that the excited-state geometry of the S1 state can deviate significantly from planarity dependent upon the substituent attached to the BODIPY core [42].

Membrane rafts are domains found within membranes containing a high concentration of sphingolipids and cholesterol [43,44,45,46,47,48]. Thermodynamically favourable packing of cholesterol with these unsaturated lipids compared with saturated lipids helps drive the formation of these domains. They are thought to compartmentalize biological functions; in particular they are considered important in cellular signaling. It is known that a phase separation occurs leading to two regions: the liquid ordered (*l*_o_) and liquid disordered (*l*_d_) regions. The first is the raft domain, while the second is considered non-raft. There has been some effort for computational approaches to be able to accurately describe the polarizable electrostatic interactions that are partially responsible for the formation of these domains. Experimentally, it has been very difficult to characterize [49], as they are resistant to detergents commonly used to extract membranes. Within raft-like bilayers, there is a strong hydrogen-bonding interaction between the hydrogen of the sphingomyelin N-H bond and the oxygen of the cholesterol hydroxyl group [50]. Traditional membrane probes do not incorporate groups allowing this interaction and as such, these probes may not anchor properly into the raft domain.

**Figure 1:**
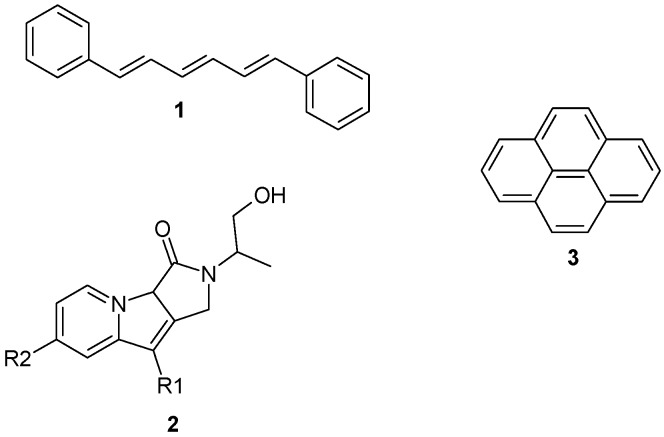
Chemical structures of the six molecules considered in this study. Molecule **2a** R_1_ = H, R_2_ = H; **2b** R_1_ = H, R_2_ = OH; **2c** R_1_ = R_2_ = OH; **2d** R_1_ = H, R_2_ = NH_2_.

**Figure 2:**
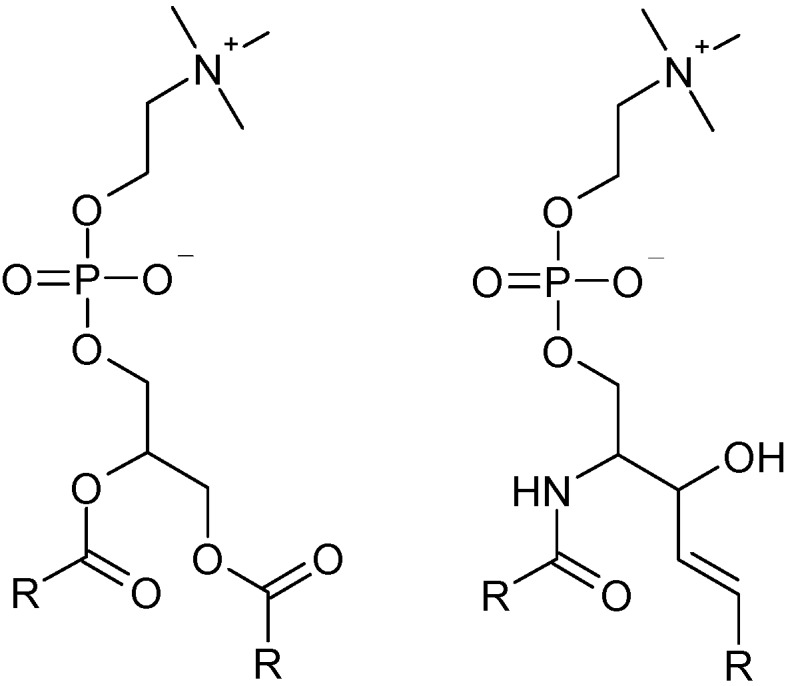
Chemical structures of the headgroup and linking region of DPPC (left) and sphingomyelin (right). In all cases, the R groups are fatty acid chains.

In this work, we consider the calculation of the six molecules shown in Figure 1 using a QM/MM approach in which the probe molecules are solvated in water and also two different lipid bilayer environments, one of which represents a “raft-like” region. Molecules **1** and **3** are commercially available, while molecules **2a**, **2b**, **2c** and **2d** are modifications of a skeletal structure that is also commercially available. The modifications considered in this study represent truncated structures, in which the *R*-groups would normally be fatty acid tails. In particular, **2c** represents a truncated structure in which the fatty acid tails most closely resemble the ester groups of the palmitoyl tails, while **2d** represents a structure corresponding more closely to the sphingolipid tails, with the amide-type linkage (Figure 2). Our aim is to understand functional groups which should allow the probes to anchor properly within a raft domain, while not having a negative effect upon the emission spectra.

## 2. Results and Discussion

Given in Table 1 are the Franck-Condon vertical excitation energies, 0-0 excitation energies and fluorescence emission energies calculated in the gas-phase and using the implicit PCM solvent. Molecules **1** and **3** show very little variation in their absorption and emission energies with respect to the dielectric environment. This is to be expected, given the hydrophobic nature of these molecules. The more hydrophilic molecules show larger shifts in general, with **2b** having the largest shift in the vertical absorption (0.11 eV) and emission energies between the gas phase and solvated in water (0.14 eV).

The vertical absorption and emission energies when explicit water molecules were included within the PCM model calculations are presented in Table 2. In all cases a small shift is observed. In particular, the emission energies are all red-shifted by ~0.1 eV with respect to the energies without explicit water present. Molecules **2a** and **2b** each have a single explicit water molecule, while **2b** and **2c** have 2 and 3 explicit water molecules, respectively. It can be concluded that when there is hydrogen-bonding to –OH groups at the R_1_ and R_2_ positions, there is no shift on the emission energies, but that any shift is due to the bulk electrostatic effect of the solvent. Comparing the results of the explicit water calculations to the PCM implicit solvent calculations implies that water hydrogen-bonded to the single –OH group from the skeletal structure of molecule **2** seems to cause a systematic shift of ~0.1 eV; this may simply reflect a better estimate of the energy when explicit solvent molecules are included rather than a continuum solvent. With this in mind, we come next to the results of each of the molecules solvated in water using a QM/MM approach. Molecules **1** and **3** did not exhibit any shift (data not shown). Molecule **2a** shows an identical shift to that observed using explicit water molecules, while **2b** and **2c** show a significant red-shift from both the PCM model and explicit water emission results. Molecule **2d** shows a similar result to that obtained using the PCM model. Molecules **2b** and **2c** both contain –OH groups on the aromatic ring and these data suggest that hydrogen-bonding at these positions may cause large shifts in the emission energy, depending on the strength of the hydrogen bond.

**Table 1:** Franck-Condon vertical excitation, 0-0 and emission energies calculated using LRC-ωPBE/6-31G(d) in the gas phase and solvated using the PCM model. All energies in eV.

Molecule	ε	Vertical	0-0	Emission	Molecule	ε	Vertical	0-0	Emission
**1**	1	3.92	3.72	3.46	**2c**	1	3.88	3.50	3.11
	6.02	3.92	3.73	3.45		6.02	3.95	3.63	3.18
	20.7	3.92	3.73	3.45		20.7	3.96	3.66	3.19
	78.4	3.92	3.73	3.45		78.4	3.96	3.67	3.19
**2a**	1	4.45	4.11	3.76	**2d**	1	4.21	3.84	3.57
	6.02	4.49	4.21	3.83		6.02	4.32	4.06	3.67
	20.7	4.50	4.24	3.84		20.7	4.33	4.10	3.69
	78.4	4.50	4.25	3.84		78.4	4.34	4.11	3.69
**2b**	1	4.27	3.93	3.58	**3**	1	4.12	4.02	3.90
	6.02	4.35	4.08	3.69		6.02	4.12	4.03	3.89
	20.7	4.37	4.11	3.72		20.7	4.12	4.04	3.89
	78.4	4.38	4.13	3.72		78.4	4.12	4.04	3.89

**Table 2:** Franck-Condon vertical excitation and emission energies calculated using LRC-ωPBE/6-31G(d) using the PCM model with additional explicit water molecules and also calculated using the QM/MM approach. All energies in eV.

	Vertical Excitation		Emission		
Molecule	PCM	Explicit Water + PCM	PCM	Explicit Water + PCM	QM/MM
**2a**	4.50	4.42	3.84	3.73	3.83
**2b**	4.38	4.31	3.72	3.63	3.49
**2c**	3.96	4.09	3.19	3.10	2.91
**2d**	4.34	4.26	3.69	3.59	3.57

**Figure 3:**
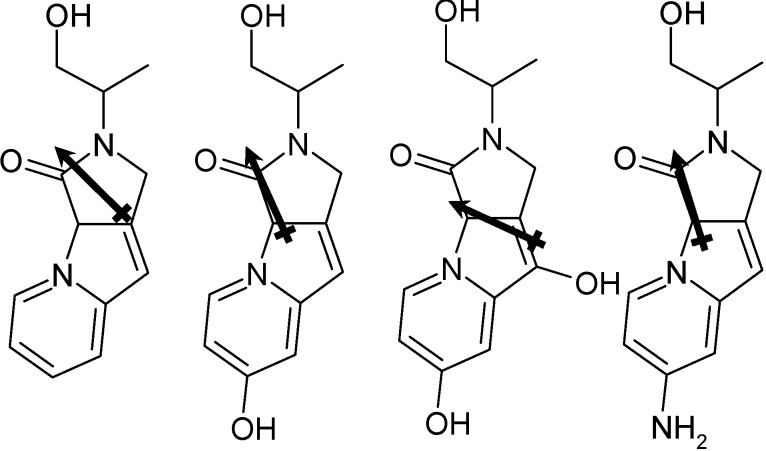
Molecules **1** and **2** orientated with respect to the membrane normal. A qualitative illustration of the ground state static dipole moment for each molecule is shown.

Within a membrane leaflet, a membrane probe should have a dipole moment which aligns with the large dipole moments of the lipid molecules. In the event that the dipole moment is opposed to these, then the probe molecule is likely to internalize. Figure 3 displays the qualitative direction of each of the dipole moments. The dipole moments are at ~30° to the orientation of the molecules as shown in Figure 3; each of the probes will therefore align with the dipole moments of the lipid molecules. While the QM/MM calculations include this information explicitly, it is worth considering the dipole moments in the gas phase so as to understand the orientation the probe will take within a lipid bilayer, and ensure that the fluorophore region of the probe is aligned with the membrane normal, such that the probe will be most effective.

Given in Table 3 are the emission energies calculated in the membrane environments using QM/MM. It is important to remember that the electrostatic potential felt by these molecules will correspond to the static potential, as we are not performing dynamics. Molecules **1**, **2a**, **2b** and **3** display almost no shift between the two membrane environments, while **2c** and **2d** display significant shifts. As molecules **1** and **3** are known to interact with the dipole potential, it is clear from these data that these probes interact with the dynamical potential. Molecules **2c** and **2d** may also interact with the dynamical potential further, but it is clear that these probe molecules, as models of potential raft-based probes, show substantial shifts between differing membrane environments, potentially allowing reporting from within membrane rafts.

**Table 3:** Emission energies of the fluorescent probes within two different membrane environments calculated with QM/MM. All energies in eV.

Molecule	DPPC	DPPC w/30 molar % Cholesterol
**1**	3.45	3.44
**2a**	3.74	3.75
**2b**	3.57	3.57
**2c**	2.99	3.47
**2d**	3.58	3.37
**3**	3.91	3.91

The results obtained here give good grounding to the use of such probes. However, one must also consider that, in expanding calculations to include more dynamical elements, the use of combined QM/MM in which the MM region is described by a polarizable force-field is a desirable approach [50,51,52,53], as the absolute value of the membrane dipole potential for the different bilayers is obtained with quantitative accuracy, whereas with a non-polarizable force-field, the difference in dipole potential is correctly predicted, but not the absolute value.

## 3. Experimental Section

Calculations were performed on the six molecules shown in Figure 2. All DFT calculations were performed using Q-Chem [54,55], while the QM/MM calculations were performed using the Q-Chem – CHARMM interface [56].

### 3.1. Gas-Phase Calculations

Ground-state gas-phase geometries were obtained using the PBE0 functional [57] with the 6-31G(d) basis set. The excited state geometries were obtained with time-dependent DFT (TDDFT) by using the long-range corrected ωPBE (LRC-ωPBE) functional [58], which was used for all energetics reported here. Previous studies utilizing TDDFT have found problems with charge-transfer states, in which the vertical excitation energies are too low. The LRC scheme partitions the electron repulsion operator into a short-range and long-range components in the evaluation of the exchange term:


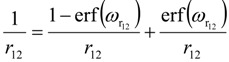
(1)

where erf is the error function and 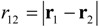. The first term on the right-hand side of equation 1 is the short-range component of the exchange and is evaluated with DFT, while the second term is the long-range component, which is evaluated with exact Hartree-Fock exchange.

### 3.2. Solvated Calculations

Ground-state and excited state geometries were obtained with the same functional and basis set combinations as for the ground-state. The first set of solvated calculations employed the polarizable continuum model (PCM) [59], with ethyl acetate (ε = 6.02), acetone (ε = 20.7) and water (ε = 78.4) used as the solvents. These solvents were selected as the dielectric constants approximately reflect the effective dielectric permittivity of the membrane interior, membrane-water interfacial region and bulk water region, respectively. For the calculation of vertical excitation energies, the reaction field was not in equilibrium with the excited state (an optical dielectric constant of 1.78 was used in all cases) [59]. For the calculation of the excited-state geometry and hence emission energies, the reaction field was in equilibrium with the excited-state, with the dielectric constant equal to that of the solvent being used (see above).

**Figure 4:**
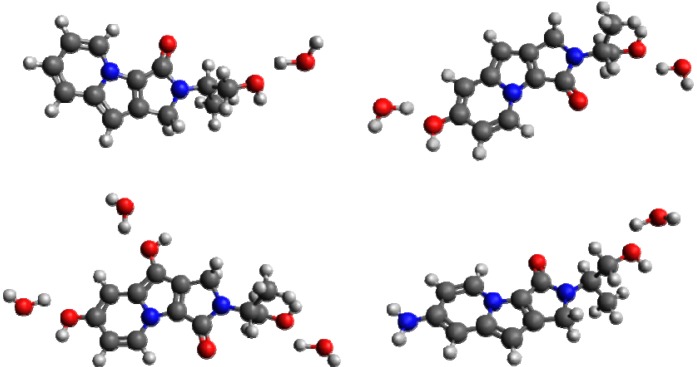
Molecules **2a**–**2d** clockwise from top-left, with added explicit water molecules.

A second set of calculations were performed for molecules **2b** and **2c** within the PCM model, with an additional explicit water molecule per –OH group of the probe molecule (see Figure 4). A geometry optimization was performed (with LRC-ωPBE/6-31G(d) ) to bring the cluster(s) to a minimum energy conformation, before the excited-state calculations were performed (as described above).

### 3.3. QM/MM Water Calculations

Each of the six molecules were solvated to a minimum depth of 10 Å with TIP3P [60] water using ad-hoc parameters of the probe molecules generated by SwissParam [61], based on data from the Merck Molecular Force-Field (MMFF). Once the molecules were solvated, the probe molecule was defined as the QM region, with the MM region comprising the water molecules. Energy minimization was performed for both the ground state and excited state of each molecule using the same functional and basis set combination as the previous calculations. The structures were considered converged using the standard CHARMM tolerances.

### 3.4. QM/MM Membrane Calculations

**Figure 5:**
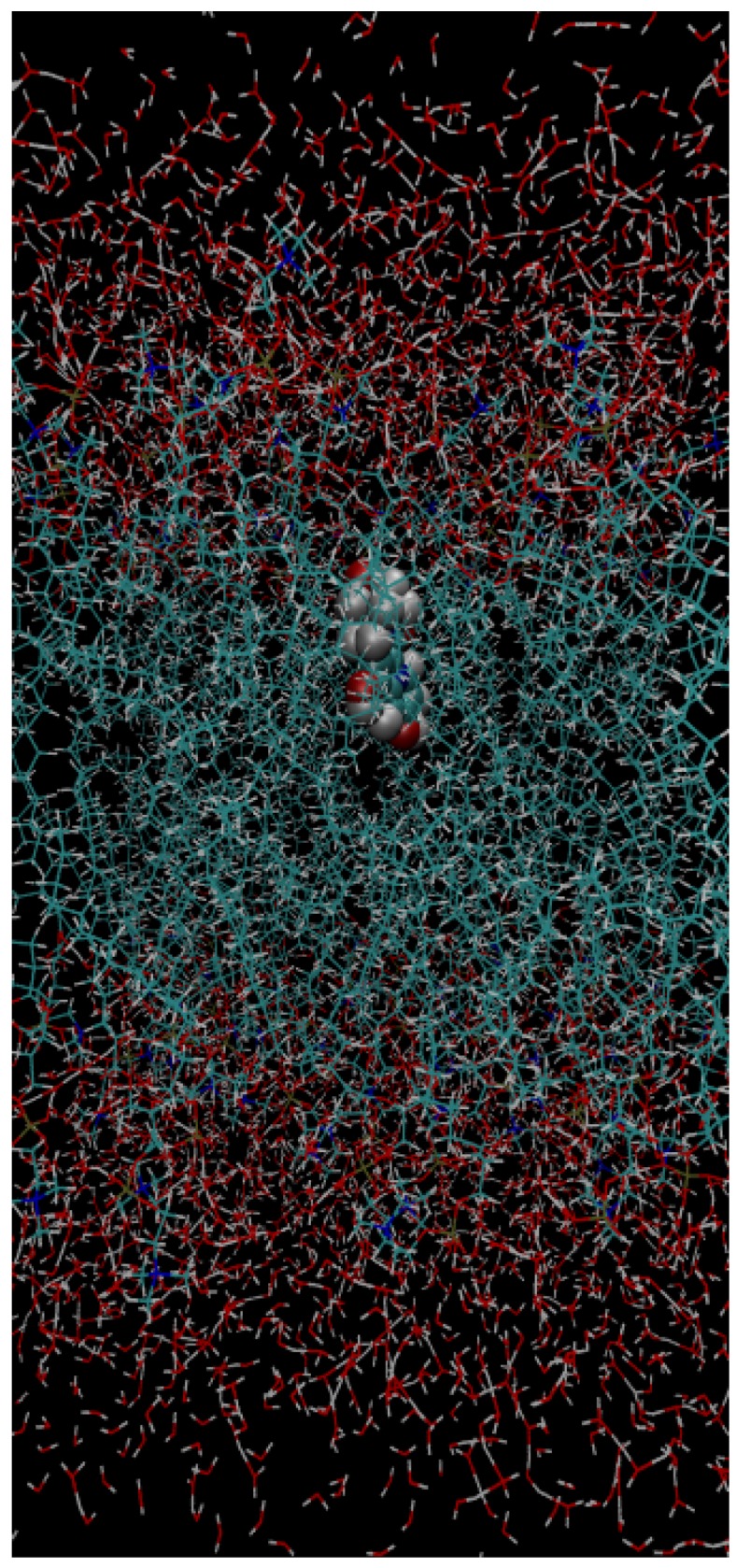
Molecule **2c** within a bilayer system for the QM/MM calculation, showing the orientation of the probes.

The ad hoc parameters for the probe molecules (above) were used in a modified form of scripts available from the CHARMM-GUI [62,63]. Two membrane systems were considered: one with a composition of pure DPPC and the second with a binary lipid composition of 30 molar % cholesterol with DPPC (see Figure 5). The system was initially equilibrated as follows: a total of 500 steps of steepest-descent minimization were initially used to remove any bad contacts, followed by a further 1,000 steps of adapted basis Newton−Raphson (ABNR) minimization, employing the CHARMM36 all-atom lipid force-field [64]. Equilibration dynamics were performed initially using a 1 fs time step for 50 ps with a nonbond cutoff of 16 Å, with the force-based switching function starting at 10 Å and eliminating all pair contributions at 12 Å. Long-range electrostatics were treated using the Particle Mesh Ewald (PME) algorithm, using a sixth order spline interpolation. Langevin temperature control was used, with damping of 10 ps^−1^. Periodic boundary conditions were used, using tetragonal symmetry. A harmonic term was used to restrain the lipid headgroups from moving too far from their starting positions. The SHAKE algorithm was used to constrain all bonds to hydrogen atoms. Further equilibration for 500 ps was performed using a 2 fs time step and a Langevin barostat with a piston period of 50 fs and a decay period of 25 fs, with the harmonic term removed. In all of these steps, the probe molecule was constrained to stay planar. The final snapshot from this equilibration stage was used as the initial geometry for the QM/MM minimization, in which the probe molecule was described using DFT (the LRC-ωPBE functional and 6-31G(d) basis set), while the CHARMM36 force-field was used to account for the lipids; TIP3P was used for the water molecules. Initially, minimization with ABNR was performed for 2000 steps, with further minimization until the change in the energy of the system fell below 0.0006 kcal mol^−1^, to be consistent with the tolerance found within the Q-Chem program.

## 4. Conclusions

A selection of contemporary and novel membrane probes, selected for their structural similarity to membrane raft components, has been investigated. Molecules **2c** and **2d**, the most representative molecules with respect to mimicking the structure of lipid molecule linkages, have shown large shifts with respect to the surrounding electrostatic environment. While considering potential probes of membrane function within raft-like domains, we suggest that these probes could offer substantial performance improvements over traditional probes when looking at fluorescence emission spectroscopy.
